# Prospective study of *POLG* mutations presenting in children with intractable epilepsy: Prevalence and clinical features

**DOI:** 10.1111/epi.12115

**Published:** 2013-02-28

**Authors:** Johanna Uusimaa, Vasantha Gowda, Anthony McShane, Conrad Smith, Julie Evans, Annie Shrier, Manisha Narasimhan, Anthony O'Rourke, Yusuf Rajabally, Tammy Hedderly, Frances Cowan, Carl Fratter, Joanna Poulton

**Affiliations:** * Nuffield Department of Obstetrics and Gynaecology, The Women's Centre, John Radcliffe Hospital, University of OxfordOxford, United Kingdom; † Department of Paediatric Neurology, Oxford Children's HospitalOxford, United Kingdom; ‡ Oxford Medical Genetics Lab, Churchill HospitalOxford, United Kingdom; § Department of Neurology, University Hospitals of LeicesterLeicester, United Kingdom; ¶ Evelina Childrens Hospital at Guy's and St. Thomas' Hospital and Kings Health PartnersLondon, United Kingdom; ** Department of Paediatrics, Imperial College, Hammersmith CampusLondon, United Kingdom

**Keywords:** Metabolic diseases, Mitochondrial diseases, Genetic epilepsies, Children

## Abstract

**Purpose:**

To assess the frequency and clinical features of childhood-onset intractable epilepsy caused by the most common mutations in the *POLG* gene, which encodes the catalytic subunit of mitochondrial DNA polymerase gamma.

**Methods:**

Children presenting with nonsyndromic intractable epilepsy of unknown etiology but without documented liver dysfunction at presentation were eligible for this prospective, population-based study. Blood samples were analyzed for the three most common *POLG* mutations. If any of the three tested mutations were found, all the exons and the exon–intron boundaries of the *POLG* gene were sequenced. In addition, we retrospectively reviewed the notes of patients presenting with intractable epilepsy in which we had found *POLG* mutations. All available clinical data were collected by questionnaire and by reviewing the medical records.

**Key Findings:**

We analyzed 213 blood DNA samples from patients fulfilling the inclusion criteria of the prospective study. Among these, five patients (2.3%) were found with one of the three common *POLG* mutations as homozygous or compound heterozygous states. In addition, three patients were retrospectively identified. Seven of the eight patients had either raised cerebrospinal fluid (CSF) lactate (n = 3) or brain magnetic resonance imaging (MRI) changes (n = 4) at presentation with intractable epilepsy. Three patients later developed liver dysfunction, progressing to fatal liver failure in two without previous treatment with sodium valproate (VPA). Furthermore, it is worth mentioning that one patient presented first with an autism spectrum disorder before seizures emerged.

**Significance:**

Mutations in *POLG* are an important cause of early and juvenile onset nonsyndromic intractable epilepsy with highly variable associated manifestations including autistic features. This study emphasizes that genetic testing for *POLG* mutations in patients with nonsyndromic intractable epilepsies is very important for clinical diagnostics, genetic counseling, and treatment decisions because of the increased risk for VPA-induced liver failure in patients with *POLG* mutations. We recommend *POLG* gene testing for patients with intractable seizures and at least one elevated CSF lactate or suggestive brain MRI changes (predominantly abnormal T_2_-weighted thalamic signal) with or without status epilepticus, epilepsia partialis continua, or liver manifestations typical for Alpers disease, especially when the disease course is progressive.

Epilepsy has both genetic and environmental causes. Recently, Ottman reported on more than 20 genes thought to be associated with idiopathic epilepsy (Ottman et al., 2010), but still these are relevant for only a small proportion of patients, and few patients have access to DNA diagnostics. This is unfortunate because genetic testing may clarify a diagnosis and allow better genetic counseling, help to optimize medication-avoiding side effects, and reduce the need for further investigation (Delgado-Escueta & Bourgeois, 2008).

The term “intractable” epilepsy has been generally used when epilepsy is difficult to treat and there has been failure of two or more first-line antiseizure medications. Instead of “intractable epilepsy,” the ILAE (International League Against Epilepsy) has recently recommended the phrase “drug resistant epilepsy” to describe the condition where two tolerated and appropriately chosen antiepileptic drugs have failed to achieve sustained freedom from seizures, with no mention of the frequency of seizures (Kwan et al., 2010). Intractable epilepsy can be associated with genetic and chromosomal abnormalities, cortical malformations, congenital and acquired central nervous system (CNS) infections, inborn errors of metabolism, hypoxic-ischemic injury, and neoplasm but in many cases it is of unknown etiology.

Several mitochondrial diseases caused by mutations in either mitochondrial or nuclear genes are characterized by “intractable” epilepsy as one of the presenting features. Epilepsy associated with mitochondrial diseases can manifest as infantile spasms; astatic convulsions; myoclonic, focal, or generalized seizures; or as epilepsia partialis continua (Canafoglia et al., 2001). It has clearly been shown that *POLG* encoding the catalytic α-subunit of mitochondrial DNA polymerase gamma, is one of the genes causing epilepsy. Patients carrying this gene frequently have epilepsy in addition to numerous other neurologic manifestations including ophthalmoplegia and ataxia (Rantamäki et al., 2001; Van Goethem et al., 2001; Lamantea et al., 2002; Van Goethem et al., 2003; Hakonen et al., 2005) and Alpers' disease (OMIM 203700), which is characterized by intractable seizures, episodic progression of neurologic symptoms, liver failure, and pharmacogenetic sensitivity to valproic acid (VPA) toxicity (Harding et al., 1995; Ferrari et al., 2005; Nguyen et al., 2005; Tzoulis et al., 2006; Uusimaa et al., 2008).

The most common mutations in the *POLG* gene are p. A467T, p.W748S, and p.G848S with carrier frequencies up to 1% in some populations (Hakonen et al., 2005; Winterthun et al., 2005; Kollberg et al., 2006). It has been shown that VPA should not to be used to treat patients with mitochondrial disease, particularly because patients with *POLG* mutations are at increased risk for VPA-induced liver failure (Ferrari et al., 2005; Gordon, 2006; Uusimaa et al., 2008; Stewart et al., 2009).

The aim of this study was to assess prospectively the prevalence of the three most common *POLG* mutations in a defined population of children with nonsyndromic intractable epilepsy, but without liver manifestation typical for Alpers disease at the presentation of their epilepsy. We have previously reported *POLG* findings in a group of children, most of whom had typical clinical features of Alpers disease (Ashley et al., 2008). We also retrospectively reviewed the notes of children with a presentation of intractable epilepsy and one of the common *POLG* mutations who did not have classic Alpers syndrome, because liver dysfunction was absent, and report on the clinical features and laboratory findings of these patients.

This study is significant for understanding and treating patients with epilepsy, because *POLG* gene mutations have a high prevalence and are hence a potentially important cause of severe intractable epilepsy. Our findings further expand the clinical phenotypes associated with *POLG* mutations. This is very important for clinical diagnostics, genetic counseling, and treatment decisions because of the increased risk for VPA-induced liver failure in patients with *POLG* mutations.

## Patients and Methods

### Setting

We performed a prospective study to estimate the minimum prevalence of intractable epilepsy caused by the most common *POLG* mutations in the pediatric population in the United Kingdom. Patients with intractable epilepsy were identified by the pediatric neurologists between the years 2007 and 2009. The catchment area was that of the John Radcliffe Hospital, Oxford, United Kingdom, which includes Oxfordshire, Berkshire, Swindon, Northampton, and Milton Keynes with a total population of 413,000 people younger than the age of 16 years (http://www.statistics.gov.uk, 2007).

The inclusion criteria for the study were (1) age 3 months to 17 years, (2) intractable epilepsy of unknown etiology (3), no liver dysfunction at presentation of epilepsy, and (4) investigation according to the prevailing practice within the pediatric neurology department. Intractable epilepsy was defined as epilepsy not controlled by two drugs with at least one or two seizures every 2 months within the previous 1 year. We assessed the family history and clinical features of the patients by means of a questionnaire, and the medical charts of the patients were reviewed. A blood sample for genetic analysis was requested from each patient fulfilling the inclusion criteria. Some of the patients found to have a *POLG* mutation also had a muscle biopsy.

### Molecular genetic analyses

Total genomic DNA was extracted from peripheral blood lymphocytes and from skeletal muscle (only from patients with verified *POLG* mutations) by standard methods. All the samples were screened for the three common *POLG* mutations, p. A467T, p.W748S, and p.G848S by polymerase chain reaction (PCR) and restriction enzyme digest (primers, restriction enzymes and conditions available on request). If one heterozygous mutation was found, the entire *POLG* coding region (23 exons, NM_002693) and intron–exon boundaries were sequenced by fluorescent dideoxy sequencing (Applied Biosystems BigDye Terminator v3.1 kit) and capillary electrophoresis (Applied Biosystems 3730 DNA Analyzer, Life Technologies, Carlsbad, CA, U.S.A.). The following resource databases were used to evaluate the pathogenicity of the *POLG* mutations: *POLG* database (http://tools.niehs.nih.gov/polg/) and splice-site prediction software (NNSPLICE 0.9, http://www.fruitfly.org/seq_tools/splice.html). Standard methods of Southern blot and long-range PCR were used for the analysis of mitochondrial DNA (mtDNA) rearrangements and real-time quantitative PCR for the analysis of mtDNA depletion (Ashley et al., 2008), in which we use age-specific normal ranges (Poulton et al., 1995; Morten et al., 2007).

### Muscle histology, histochemistry, and biochemistry

Muscle biopsies were obtained according to standard procedures. Muscle histology, histochemistry, and measurement of the activities of respiratory chain complexes I–IV were performed by standard methods at the participating centers.

### Ethical considerations

Once the diagnosis of intractable epilepsy was made, the pediatric neurology consultants/registrars explained the purpose of the DNA analyses to the parents. The samples from children were studied after obtaining informed parental consent. The proband's parents' blood samples were examined after obtaining written informed consent and parental permission. The research protocol was approved by the National Research Ethics Service (United Kingdom).

## Results

### Prevalence of the common *POLG* mutations among children with intractable epilepsy

We identified five children with one of the three most common *POLG* mutations among the prospective cohort of 213 pediatric patients with nonsyndromic intractable epilepsy without known liver problems in the population as defined above.

### Screening of the three common *POLG* mutations and subsequent *POLG* sequencing

The five prospectively identified children (Table 1, Data S1, patients 1–5) had the following combinations of *POLG* mutations: (1) p.G848S (c.2542G>A) in exon 16 in *trans* with p.P587L (c. 1760C>T) and p.P589T (c.1765C>A) in exon 10, patient 1; (2) homozygous p.A467T (c.1399G>A) in exon 7, patient 2; (3) p.A467T (c.1399G>A) in exon 7 in *trans* with p.R417T (c.1250G>C) in exon 6, patient 3; (4) p.W748S (c.2243G>C) in exon 13 in *cis* with a polymorphism p.E1143G (c.3428A*>*G) in exon 21 and in *trans* with p.G1205E (c.3614G>A) in exon 22, patient 4; and (5) p.A467T (c.1399G>A) in exon 7 in *trans* with p.G848S (c.2542G>A) in exon 16, patient 5.

**Table 1:** Clinical findings of *POLG* patients (1–5) with intractable epilepsy without liver involvement at presentation (prospective cohort)

Patient number/Gender	*POLG* mutation	Family history	Other clinical symptoms	Epileptic seizure type, age at onset	EEG findings	Brain MRI: Initial MRI/repeat MRI	Previous VPA treatment	Liver dysfunction,age at onset	Age at death/current age
1/F	p.[G848S]+[P587L; P589T]	−	Severe hypotonia, poor feeding, developmental delay	Neonatal seizures, 0 days	General slowing with right frontal and temporal sharp waves	Normal/Demyelination	No	Yes, 4 months	Death at 4 months
2/M	p.[A467T]+ [A467T]	−	Ataxia from 11 years, nystagmus, cataplexy, behavioral problems, low weight	EPC with acute onset, 15 years; focal left-sided epilepsy with secondary generalization	General slowing with right-sided emphasis	High T_2_ signal intensities in right thalamic and cortical regions	No	No	Current age, 19 years
3/M	p.[A467T]+ [R417T]	+ Father died from HOCM (heterozygous for p. R417T)	Severe encephalopathy, severe visual impairment after SE (cortical blindness)	SE, 22 months, followed by EPC, focal epilepsy with secondary generalization	Diffusely slow EEG with a very active left frontal spike and wave pattern	Normal/bilateral T_2_ high signal intensities in thalamic regions	Yes	No	Death at 2.5 years
4/M	p.[W748S; E1143G]+[G1205E]	+ Mother with moderate learning difficulties (LD), siblings also with LD	Sleeping difficulties, hyperactivity, autistic features, developmental delay	Drop attacks, 7 years, photosensitive focal generalized seizures and atypical absences and myoclonia	Multifocal spikes over both frontotemporal regions	High T_2_ signal intensities in dentate nuclei and in thalamic regions	Yes	No	Current age, 12 years
5/F	p.[A467T]+ [G848S]	−	Normal development prior to SE followed by general hypotonia, upper limb weakness, fluctuating right hemiparesis and visual impairment	SE, 17 mo, followed by EPC, focal left-sided epilepsy, viral illness prior to SE		High T_2_ signal intensities in right parieto-occipital cortex and putamen/high T_2_ signal intensities in right parietal and left frontoparietal cortex	No	No	Death at 2 years 1 month

SE, status epilepticus; EPC, epilepsia partialis continua; HOCM, hypertrophic obstructive cardiomyopathy; VPA, valproic acid; n.a., not applicable.

The three retrospectively identified patients (Table 2, Data S1, patients 6–8) had the following combinations of *POLG* mutations: (6) p. A467T (c.1399G>A) in exon 7 in *trans* with p.G848S (c.2542G>A) in exon 16, patient 6, (7) homozygous p.W748S (c.2243G*>*C) in exon 13 with a homozygous polymorphism p.E1143G (c.3428A*>*G) in exon 21, patient 7, and (8) p. A467T (c.1399G>A) in exon 7 in *trans* with p.L966R (c.2897T>G) in exon 18, patient 8.

**Table 2:** Clinical findings of *POLG* patients (6–8) with drug resistant epilepsy (retrospective cohort)

Patient number/Gender	*POLG* mutation	Family history	Other clinical symptoms	Epileptic seizure type, age at onset	EEG findings	Brain MRI: Initial MRI/repeat- MRI	Previous VPA treatment	Liver dysfunction, age at onset	Death age/Current age
6/F	p.[A467T]+ [G848S]	−	Global develop-mental delay, muscular hypotonia, severe encephalopathy with deterioration after SE	SE (18 months) followed by multiple seizures	Very disordered background activity with epileptiform discharges with right temporal predominance	Normal/basal ganglia changes and brain atrophy	No	No	Death at 2 years
7/F	p.[W748S; E1143G]+ [W748S; E1143G]	−	Several SEs, bilateral cortical blindness, nystagmus, lower limb weakness	SE (16 year) 12 months after the first focal seizure, EPC, focal left-sided epilepsy with secondary generalization	Epileptiform activity predominantly on the right temporoparietal region	Normal/high T_2_ signal intensities in thalamic, occipital and cerebellar regions	Yes	Minimal, 18 years	Death at 18 years
8/F	p.[A467T]+[L966R]		Developmental regression from 15 months	SE (18 months), viral illness prior to SE	Burst suppression pattern	Cerebral edema, bilateral high T_2_ signal intensities in thalamic regions	No	Yes, 18.5 months	Death at 19 months

SE, status epilepticus; EPC, epilepsia partialis continua; VPA, valproic acid; n.a., not applicable.

In three patients, both mutations were located in the linker domain of DNA polymerase gamma, and in five patients, one mutation was located in the linker domain and the other mutation in the polymerase domain.

### Clinical characteristics of the patients with the *POLG* mutations

Clinical histories from the eight patients are detailed in Data S1. The clinicopathologic and laboratory findings of patients 1–5 are summarized in Tables 1 and 3, and the data on retrospectively identified patients 6–8 in Tables 2 and 3.

**Table 3:** Laboratory findings of patients with intractable epilepsy associated with *POLG* mutations

Patient number	*POLG* mutation	P-lactate/CSF lactate	Liver function tests	Histochemistry of muscle and liver	Mitochondrial respiratory chain function in muscle or liver	MtDNA analysis of muscle
1	p.[G848S]+[P587L; P589T]	2.7–4.9/4.6	ALT ↑, γGT ↑, Bil ↑, PT ↑, APTT ↑	Muscle: Increased Lipid	Muscle: Normal	MtDNA depletion (17% of normal mean)
2	p.[A467T]+[A467T]	Normal/Normal	Normal	n.d.	n.d.	n.d.
3	p.[A467T]+[R417T]	Normal/2.6	Normal	Muscle: Normal	Muscle: Normal	Normal
4	p.[W748S; E1143G]+ [G1205E]	2.5/Normal	Normal	Muscle: Normal	Muscle: Normal	Normal
5	p.[A467T]+[G848S]	Normal/2.8	Normal	n.d.	n.d.	n.d.
6	p.[A467T]+ [G848S]	Normal/2.5	Normal	Muscle: Myopathy with RRF	Muscle: Normal	n.d.
7	p.[W748S;E1143G]+ [W748S; E1143G]	Normal/Normal	γGT ↑ ALT ↑	Muscle: Normal except for increased lipofuscin	Muscle: Normal	Normal
8	p.[A467T]+[L966R]	2.0–4.0/Normal	ALT ↑	Muscle: Normal Liver: microvascular steatosis with oncocytosis with hepatocytes containing large and small droplet lipids	Muscle: Decreased COX	Normal

ALT, alanine aminotransferase; γGT, gamma glutamyltransferase; Bil, bilirubin; PT, prothrombin time; APTT, activated partial thromboplastin time; RRF, ragged red fibers; COX, cytochrome oxidase; n.d., not done.

Plasma lactate, normal range is 0.6–2.4 mm, CSF lactate, normal range is 0.9–2.4 mm.

**Figure 1:**
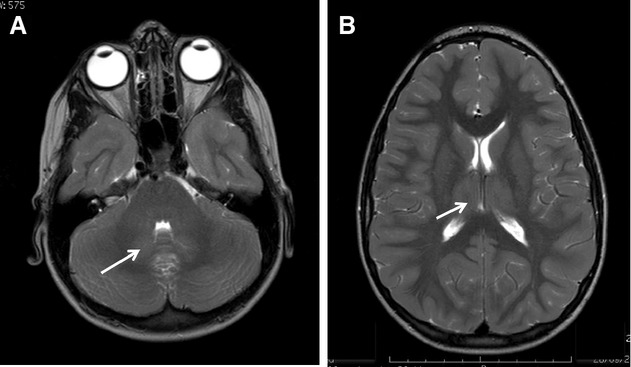
Increased T_2_ signal intensities in dentate nuclei and thalamic regions in brain magnetic resonance imaging (MRI) associated with *POLG* mutations. Axial T_2_-weighted MR images from patient 4, who had p.W748S;E1143G + G1205E mutations. There is mildly increased signal intensity (SI) in the region of the dentate nuclei (**A**, arrow) and diffuse high SI in both thalami (**B**, arrow). Increased SI in the same areas was also seen on the fluid-attenuated inversion recovery (FLAIR) images but no abnormal findings were detected on the T_1_-weighted images. No other lesions were seen in this patient and the findings were very similar between the scan at presentation (shown) and a scan done 3 months later.

In the prospective cohort the first epileptic seizures manifested at a mean age of 3.7 years (range 5 h to 15.3 years). The first clinical symptoms were epileptic seizures (patients 1, 3, and 5), ataxia (patient 2), and behavioral and developmental problems with autistic features (patient 4). The seizures included neonatal seizures (one patient), drop attacks and atypical absences with myoclonia (one patient), focal epileptic seizures with/without secondary generalization (five patients), status epilepticus (two patients), and epilepsia partialis continua (three patients). Brain MRIs revealed demyelination (1), increased T_2_ signal intensities in thalamic regions (3) or cortical areas (2), or in dentate nuclei/putamen (2) (Fig. 1), but the initial brain MRI was reported as normal in two of five cases. EEG findings included general slowing with focal temporal and frontal spikes and waves, but none of the patients had occipitally located seizure activity. None of the patients had liver dysfunction at the time of presentation with epilepsy, and four did not develop it even though two were treated with VPA (patients 3 and 4). Patient 1 developed acute fatal liver failure at 4 months of age without previous VPA treatment.

**Figure 2:**
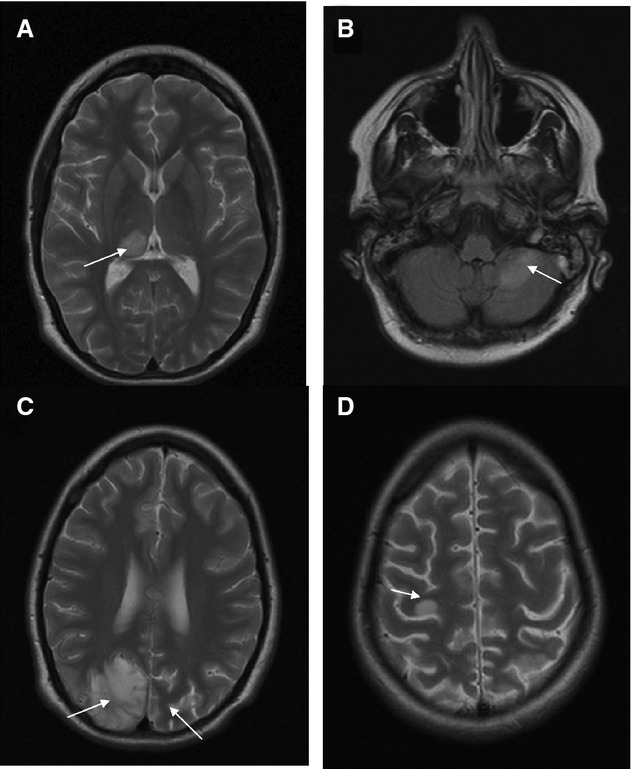
Progressive changes in brain MRI with continued fitting associated with *POLG* mutations. The brain MRI at presentation with the first focal epileptic seizure of patient 7 with a homozygous p.W748S/p.W748S was normal (not shown). On admission 1 year later a clearly circumscribed region of abnormal high signal intensity (SI) was seen in the right posterior thalamus on T_2_-weighted and FLAIR images (**A**, axial T_2_-weighted image shown at the level of the basal ganglia) and abnormal low SI on T_1_-weighted imaging (not shown). Lesions of similar SI were seen in two sites in the left cerebellar hemisphere (**B**, axial FLAIR image). Two months later, bilateral parietal-occipital lesions involving the white matter and cortex (right much more than left) were seen (**C**, axial T_2_-weighted image) with improvement in the previous abnormalities without atrophy, although the abnormal SI in the left thalamus remained obvious. A further scan 3 months later showed new lesions in the cortex (**D**, axial T_2_-weighted image, arrow).

In the retrospective cohort (Data S1, patients 6–8) the first epileptic seizures manifested at a mean age of 6.3 years (range 1.5–16 years). The seizure types included focal seizures with secondary generalization (3), status epilepticus (3), and epilepsia partialis continua (1). In addition, presenting symptoms included developmental regression, hypotonia and lower limb weakness, bilateral cortical blindness, and nystagmus. Their brain MRI scans revealed high-signal intensities on T_2_-weighted images in thalamic, occipital, and cerebellar regions in addition to basal ganglia changes and brain atrophy, but the initial brain MRI was normal in two of three cases (Fig. 2). Electroencephalography (EEG) findings included burst suppression pattern or very disordered background activity and epileptiform discharges with temporal prominence. These three patients had a progressive disease course and died at the mean age of 6.6 years (range 19 months to 18 years). One patient not treated with VPA developed liver failure and another patient presented with a minimal liver dysfunction having had VPA treatment.

The mean age at onset of epileptic seizures in all eight patients was 3.81 years (range, 5 h to 16 years). Patients manifested with several types of seizures, but the most common ones were focal seizures with or without secondary generalization (eight patients), which led to status epilepticus in five of eight cases and epilepsia partialis continua in four of eight cases. The most common EEG findings were temporal (4/7) and/or frontal (3/7) epileptiform discharges and general slowing (3/7). The most common brain MRI findings were high signal intensities in thalamic regions on T_2_-weighted images (5/8, Fig. 1); in four cases the initial brain MRI at the onset of symptoms was normal. The disease was fatal with a rapidly progressive course in six cases; the mean age at death was 3.5 years (range 4 months to 18 years), and only 8.5 months (range 4 to 24 months) after the first epileptic seizure and 9 months after the first symptom. The patients with homozygous p. A467T/p.A467T and a compound heterozygous p. W748S/p.G1205E mutation are alive currently at 19 and 12 years, respectively.

### Laboratory findings of patients with *POLG* mutations

Muscle biopsies were available in six of the eight patients; respiratory chain enzyme activities were normal in 5 and muscle histology and histochemistry were normal in three samples (Table 3). One patient (patient 8) had decreased (cytochrome oxidase) activity in muscle with normal muscle histology, but microvascular steatosis with oncocytosis was found in the liver. In patient 1, mtDNA depletion in muscle (with 17% of mtDNA of normal mean) was found together with increased lipid, and patient 6 presented with myopathy and ragged red fibers (RRFs) in muscle. Plasma lactate was elevated in three of eight patients (range 2.0–4.9 mm, normal <1.8 mm) and CSF lactates were slightly elevated in four of eight patients (range 2.5–4.6, normal <2.4 mm). Liver function tests were abnormal in three patients.

## Discussion

We found that the minimum prevalence of the three most common *POLG* mutations either as homozygous or compound heterozygous state was 2.3% among a prospective cohort of 213 children with intractable epilepsy without liver manifestation at presentation of epilepsy. Epilepsy is one of the most common neurologic disorders, affecting 1–5% of the population worldwide (World Health Organization estimate). In up to 40% of the patients, genetic factors have been implicated (Elmslie et al., [Bibr b11]). About 20–30% of children have been reported to meet criteria for intractable epilepsy early in the course of their epilepsy (Ko & Holmes, 1999; Aicardi, 2004; Berg, 2009). Because the total United Kingdom population was 61,792,000 in 2009 of which 19% (11,740,480) was younger than the age of 16 years (http://www.statisctics.gov.uk), we estimate there to be about 117,000 children with epilepsy (1%). Assuming that 20–30% of these patients have intractable epilepsy, there would be about 24,000–35,000 children with drug-resistant seizures in the United Kingdom. Most intractable epilepsies in children (about 50%) are caused by perinatal brain damage (Sillanpää, 1993; Chawla et al., 2002),. Other etiologies include cortical malformations, various congenital disorders including chromosomal abnormalities, congenital and acquired CNS infections, brain tumors, and defined metabolic diseases, and the remaining 20–30% are at present considered idiopathic in etiology. Assuming a genetic etiology for this latter group with nonsyndromic childhood-onset intractable epilepsy, we speculate that there could be about 110–240 children in the United Kingdom with intractable epilepsy associated with at least one of the three common *POLG* mutations as homozygote or compound heterozygote state.

In this study, we did not perform full *POLG* sequencing of all the 213 DNA samples whereby we might have identified more patients with other pathogenic *POLG* mutations. However, our previous study (Ashley et al., 2008) suggests that by screening these three common mutations, we should detect the majority of patients with intractable epilepsy related to *POLG* mutations. Unpublished data from our entire cohort of patients with autosomal recessive *POLG* mutations indicate that three mutations (p.A467T, p.W748S, and p.G848S) account for approximately 50% of all mutations in our referral population. Consequently, approximately 75% of patients are at least heterozygous for one of these mutations and so will be identified by a primary test for just these three common mutations. This predicted proportion of 75% is borne out by our data (31/43 patients have at least one of the common three mutations [unpublished data]).

In all, we identified eight patients with intractable epilepsy without liver manifestations at presentation of their epilepsy associated with the following combinations of *POLG* mutations: p.[G848S]+[p.P587L;p.P589T], p.[A467T]+[A467T], p.[A467T]+[R417T], p.[W748S]+[G1205E], p.[A467T]+[G848S], p.[W748S]+[W748S], and p.[A467T]+[L966R]. All these mutations have been previously reported as pathogenic mutations in *POLG* database (http://tools.niehs.nih.gov/polg) except for the two novel nucleotide changes, namely c.1765C>A (p.P589T) and c.3614G>A (p.G1205E). None of these patients had mutations in the catalytic regions of both alleles. In three patients both mutations were in the linker region, and the remainder had one catalytic and one linker mutation. Consistent with our previous study, their clinical and cellular phenotypes were milder than patients with two catalytic mutations (Ashley et al., 2008). The novel combinations of *POLG* mutations were p.G848S (c.2542G>A) in *trans* with p.P587L (c. 1760C>T) and p.P589T (c.1765C>A) in a patient with intractable neonatal seizures followed by fatal liver failure in infancy which developed into an Alpers disease phenotype (patient 1) and p.W748S (c.2243G>C) in *trans* with p.G1205E (c.3614G>A) in a patient with childhood-onset intractable epilepsy with behavioral problems and autistic features without liver manifestations by the age of 12 years (patient 4). Furthermore, it is worth mentioning that Patient 4 presented first with a long-standing autism spectrum disorder before seizures emerged. Therefore, autistic features can also be associated with *POLG* mutations further expanding implications for *POLG* testing. The nucleotide change c.1765C>A in exon 10 leads to substitution of the same amino acid p.P589, as the previously reported mutation c.1766C>T (p.P589L) in *cis* with p.P587L and in *trans* with p.W748S associated with Alpers phenotype (Ashley et al., 2008). Similarly, the amino acid substitution p.G1205E. A change at the same residue (p.G1205A) caused by heterozygous mutations (c.3614G>C) was associated with retinitis pigmentosa, hearing loss, and failure to thrive (Wong et al., 2008). It is likely that the *POLG* c.3614G>A variant is a disease-causing mutation, since (1) glycine to glutamate is a nonpolar to acidic amino acid substitution, (2) glycine at position p.1205 is highly conserved across species, and (3) this variant affects an amino acid within the functionally important polymerase domain of the protein.

The common *POLG* p.A467T mutation has previously been reported as a homozygous mutation in ataxia, sensory neuropathy, dysphagia, epilepsy, Alpers disease, progressive external ophthalmoplegia (PEO), and sensory ataxic neuropathy, dysarthria, and opthalmoparesis (http://tools.niehs.nih.gov/polg) and in *trans* with p.L966R (Nguyen et al., 2006). We found *POLG* p.A467T mutation in *trans* with p.R417T associated with intractable epilepsy with status epilepticus as the first manifestation of the disease without any liver symptoms leading to severe epileptic encephalopathy and death at the age of 2.5 years. The following evidence suggest that p.R417T (c. 1250G>C) is pathogenic: (1) COMPUTER software (http://www.fruitfly.org) predicts that c.1250G>C significantly reduces the strength on the intron 6 splice donor site, and so may lead to aberrant splicing; (2) arginine to threonine is a nonconservative amino acid substitution (charged polar to uncharged polar); (3) arginine at codon 417 is highly conserved across species (human to fly); and (4) this variant affects an amino acid within the functionally important exonuclease domain of the protein. Although this study was still ongoing, the cellular phenotype in fibroblasts of this patient was reported by Ashley et al. (2008).

The second common *POLG* mutation, p.W748S, usually found in *cis* with p.E1143G, has been identified in the homozygous state or compound heterozygous with other *POLG* mutations in patients with various clinical manifestations including early onset Alpers disease, PEO, ataxia, sensory neuropathy, PEO, and dysphagia (http://tools.niehs.nih.gov/polg). In addition to these, we here report a new phenotype with early onset behavioral problems with autistic features and developmental delay followed by childhood-onset intractable epilepsy associated with the genotype p. [W748S]+ [G1205E], but without other characteristic features of mitochondrial diseases.

The third common *POLG* p.G848S mutation has been reported with Alpers phenotype in different combinations with the mutations p.T251I, p.A467T, p.Q497H, p.P587L, or with p.W748S and p.E1143G (http://tools.niehs.nih.gov/polg). Our patient with the genotype p.[A467T]+[G848S] presented with epilepsia partialis continua and status epilepticus prior to a viral infection in a previously healthy child with normal development and in another patient with global developmental delay, muscular hypotonia, and intractable epilepsy with severe encephalopathy. Both these patients manifested with clinical deterioration after the status epilepticus at the ages of 17 and 18 months and with rapid progression leading to death at the age of 2 years. In these patients, brain MRI revealed characteristic features for mitochondrial diseases due to *POLG* mutations including cortical signal intensities, brain atrophy, basal ganglia changes, but without liver manifestation as has been the case in some Alpers patients (Ferrari et al., 2005). Patient 1, compound heterozygous for p.G848S and p. [P587L; P589T], presented with neonatal-onset intractable seizures followed by fatal liver dysfunction, which differed from the previously described patient with p.[W748S]+[P587L;P589L] who had juvenile-onset (17 years) epilepsy and movement disorder (Ashley et al., 2008).

The clinical diseases caused by *POLG* mutations are enormously variable in severity, ranging from mild ataxia and chronic PEO to severe Alpers disease, but with some phenotype–genotype correlation (Ashley et al., 2008). Most of our eight patients lacked the characteristic features of mitochondrial diseases such abnormal skeletal muscle mtDNA, histology, and biochemistry. Only some of the *POLG* patients have increased plasma lactate levels and increased lactate–pyruvate ratio, or increased CSF lactate. The common brain MRI findings in *POLG* patients include lesions of high signal intensity on T_2_-weighted imaging in the thalamus, cortical areas or cerebellar white matter, or atrophy of the cerebellum or cerebellar vermis (Rantamäki et al., 2001; Van Goethem et al., 2004; Wolf et al., 2009), but brain MRI can be normal especially on presentation. Abnormal brain MRI findings may also disappear on repeated MRI scanning as in our patient 7 (Fig. 2A–D). However, seven of our eight patients with *POLG* mutations had either slightly raised CSF lactate or MRI changes at the presentation of their intractable epilepsy.

The location of the mutation in *POLG* gene and the type of the mtDNA mutation determine at least partly the clinical phenotype. Milder disease may be caused by linker region mutations in *POLG* and multiple mtDNA deletions, whereas the most severe form of disease is typically associated with a catalytic domain mutation in both alleles, resulting in severe mtDNA depletion (Ashley et al., 2008). MtDNA depletion has been documented in liver, muscle or brain in patients with different *POLG* mutations (Poulton et al., 1994; Naviaux et al., 1999; Naviaux & Nguyen, 2004; Tesarova et al., 2004; Ferrari et al., 2005; Uusimaa et al., 2008), and we were among the first to insist on using age-adjusted normal ranges (Poulton et al., 1995; Morten et al., 2007; Poulton & Holt, 2009). The clinical features of patients 6–8 with an early or juvenile onset of Alpers disease were typical for the disease phenotypes, which have been associated with p.A467T and/or p.W748S mutations, with onset before 3 years of age for compound heterozygotes and with a later onset, typically after 7 years, in homozygotes (Naviaux et al., 1999; Di Fonzo et al., 2003; Naviaux & Nguyen, 2004; Davidzon et al., 2005; Ferrari et al., 2005; Nguyen et al., 2005, 2006; Uusimaa et al., 2008). In addition, patient 1 developed fatal liver failure and thus developed typical features for early infantile onset Alpers disease. Of interest, the clinical features of our patient 4 with autistic traits and developmental delay have been associated with mitochondrial dysfunction as shown by abnormalities in muscle histology, mitochondrial respiratory chain dysfunction, and large-scale mtDNA deletions (Fillano et al., 2002). *POLG* gene analysis, however, was not performed in these patients.

A wide variety of epileptic seizures have been reported as the first recognized symptom in 53% of patients with a variety of mitochondrial diseases (Canafoglia et al., 2001), the most common types being intractable or recurrent status epilepticus, myoclonic seizures, infantile spasms, and epilepsia partialis continua (El Sabbagh et al., 2010). In a recent publication on 19 patients with the two most common *POLG* mutations, p.W748S or A467T, 13 (76%) had epilepsy, which was an early and defining feature of the disease with a poor prognosis (Engelsen et al., 2008). Typically Alpers patients have both simple and complex focal seizures, clonic and/or myoclonic seizures with epilepsia partialis continua, frequent convulsive status epilepticus, and secondary generalized or multifocal epilepsy with a focal occipital predilection (Engelsen et al., 2008; Wolf et al., 2009). Intractable status epilepticus may even be the first symptom of the disease in some patients (Horvath et al., 2006; Tzoulis et al., 2006; Engelsen et al., 2008; Uusimaa et al., 2008; Wolf et al., 2009) as we found in four of eight patients in this study. EEG findings for *POLG* disease include early predominance of epileptiform discharges over the occipital region (Engelsen et al., 2008; Wolf et al., 2009), but the EEG findings can vary as we found. Acute liver failure after the administration of VPA is common among patients with Alpers disease, but liver dysfunction has also been described in *POLG* patients without VPA administration (Ferrari et al., 2005; Uusimaa et al., 2008), as was the case in two of our patients (patients 1 and 8). A recent review described four *POLG* patients (age range 3 to 18 years) given VPA for intractable partial seizures followed by liver failure where the time from VPA exposure to liver failure was 2–3 months, posing the question of whether *POLG* sequencing should be considered prior to VPA treatment (Saneto et al., 2010).

In conclusion**,** *POLG* mutations have a high prevalence and are hence a potentially important cause of severe intractable epilepsy. Rapid PCR-based screening for common *POLG* mutations has recently become available in United Kingdom diagnostic laboratories and should become routine in patients with intractable epilepsy. Our results emphasize screening of the common *POLG* mutations and *POLG* sequencing in any child or adolescent who presents with intractable seizures and at least one raised CSF lactate (or brain magnetic resonance spectroscopy lactate) or suggestive brain MRI changes (with thalamic predominance) with or without status epilepticus, epilepsia partialis continua or liver manifestations typical for Alpers disease, especially when the disease course is progressive.

## Supporting Information

**Data S1.** *POLG* patients (1-5) identified from the prospective cohort of children with intractable epilepsy without liver involvement at presentation.
