# Supplementary material for: Prospective study of POLG mutations presenting in children with intractable epilepsy: Prevalence and clinical features
Source: Epilepsia. 2013 Feb 28;54(6):1002–11. doi: 10.1111/epi.12115 (PMC3757309; doi:10.1111/epi.12115)
Supplement: Supplementary file 1 [file epi0054-1002-SD1.doc]

**Supplementary Data**

***POLG1 patients (1-5) identified from the prospective cohort of children with intractable epilepsy without liver involvement at presentation***

*Patient 1* was a girl, who was born at term by normal delivery and was the second baby of healthy parents (birth weight 3.49 kg, head circumference 34 cm, Apgar scores 7 at 1 minute and 10 at 5 minutes). The baby was admitted to the special care unit at the age of 5 hours old, because of seizures (tonic seizures with fisting, arching back and clonic movements), hypotonia and poor feeding. When phenobarbitone treatment was started, her episodes of fisting decreased, although she had a few further episodes in the first couple of days. On day 3 and day 4 she was found to have significant head lag, significant hypotonia, absent Moro, poor sucking and weak cry. In the interictal EEG at the age of 6 days (on phenobarbitone treatment) the background rhythm with no significant asymmetries was found, no significant abnormalities and no epileptiformic activity. On day 28 she was sent home, but when weaning her off the phenobarbitone the seizure episodes reoccurred. After restarting the phenobarbitone medication, seizures stopped, but the baby was very sleepy and significantly hypotonic. At the age of 6 weeks she had a prolonged generalised seizure, lasting 10-15 minutes, and because of that phenobarbitone was changed to carbamazepine. On her referral to a paediatric neurologist at the age of 3 months she was found to have severe hypotonia, poor feeding and developmental delay. In EEG recordings at the age of 2 months 1.5-2 Hz delta activity dominated throughout all regions intermixed with 4-5 Hz theta activity, and sleep spindles (11-13 Hz) were seen bilaterally. In addition, frequent sharp waves were seen over the right anterior to mid temporal regions. She was admitted to hospital at the age of 4 months due to an episode of vomiting. During this admission she was noted to have abdominal distension with hepatosplenomegaly and abnormal liver function. She received i.v. cefotaxime, amoxicillin, acyclovir, fluconazole and fibrinogen, and was admitted to the paediatric intensive care unit. Cardiac EHCO was normal, she was sedated with i.v. morphine and i.v. midazolam. EEG showed evidence of encephalopathy due to medications, metabolic disorder and underlying brain dysfunction. Carbamazepine medication was changed to levetiracetam due to abnormal liver function after which there were no seizures. Renal functions were normal, but she was jaundiced and had hepatosplenomegaly with normal echotexture, normal spleen and kidneys. She had persistent lactic acidosis since admission, and she died due to liver failure at the age of 4 months.

*Patient 2* is currently an 18 year-old boy, which had some impairment of mental concentration from the age of five years, but otherwise normal development until the age of 11 years when he presented with mild ataxia. At the age of 15 years 4 months he presented with twitching of the left toes followed by *epilepsia partialis continua* which was restricted to the left side, starting in the left foot and then mainly occurring in the left trunk and leg with some localised twitching also in the left neck. A few days earlier he had fallen from his motor cycle. At the hospital he required two long periods of ventilation with nasogastric feeding because of severe focal epilepsy with generalized epileptic seizures. While medicated on intensive care he developed complete upgaze failure with horizontal nystagmus. Abdominal ultrasound showed hepatosplenomegaly, but liver function tests were normal. EEG revealed generalized slowing in the theta range both anteriorly and posteriorly with right sided emphasis. Brain MRI scan showed a small focus of increased signal without enhancement in the right motor cortex and a larger focus in the right thalamus. He was treated with phenobarbitone. He soon appeared to develop cataplexy, so that with anger and pain he fell from standing or had persistent trunk flexion in the seated position. At the same time aggressive behavioural episodes developed, but he improved on reducing phenobarbitone. 7 weeks from the onset of seizures he had gait ataxia, but he could walk very short distances unaided. Abnormal eye signs largely resolved. He was found to be areflexic with toe and ankle dorsiflexion weakness. Due to poor dietary intake domperidone medication was started. Currently he has focal left -sided epilepsy with seizure manifestations almost daily, particularly with anger. His epilepsy became more active after levetiracetam was inadvertently omitted just before and after hospital discharge for nearly one week, providing evidence of efficacy of this drug to his seizures. Current medication includes clonazepam, phenobarbitone, oxcarbazepine, and levetiracetam. In addition to these, he has citalopram treatment for possible cataplexy which had caused injury. On examination at the age of 16 years he was very thin (BMI 17) and had mild dementia and behavioural disorders, nystagmus, and ataxia. There was no known family history, and his younger sister has a different father.

*Patient 3* was a boy, who presented with his first epileptic seizures at the age of 22 months with a mild febrile disease, but with a severe encephalopathy comprising focal and generalized seizures leading to *status epilepticus* requiring intensive care support. Head CT and subsequent brain MRI scans were both normal. Glucose, liver function, amino and organic acids were all normal at that time, but CSF lactate was slightly elevated (2.6 mmol/l). Seizure control was achieved (not with valproate, but with levetiracetam and phenobarbitone) and he returned to almost normal except for severe visual impairment which could not be explained by his EEG findings. Eye fundoscopy and ERG were normal, but VEP was slightly delayed (right and left). Renal function tests, ECG and cardiac ECHO were normal. Then he developed *epilepsia partialis continua* (persistent focal seizure affecting left side of the body), and he died at the age of 2.5 years due to intractable epilepsy, but without liver dysfunction. He has a brother (about 3 yrs) and a sister (about 19) without any known health problems. Since then his father (Algerian) has died due to hypertrophic obstructive cardiomyopathy, but not known to have any cardiac problems prior his acute cardiac presentation.

*Patient 4* is a boy born at term by spontaneous vaginal delivery. Following the birth he was recommended for foster placement due to social circumstances. His Mother had a reported IQ of around 30 and a maternal sister had reported learning difficulties. There were five older half-siblings who had learning difficulties. Since the neonatal period he had presented with behavioural difficulties and sleep problems. Motor development was normal and he walked at 10 months. His first words appeared around 10 months and he started putting words together prior to 2 years.There was a reported period of developmental regression between the age of 2-2 ½ years when a diagnosis of autistic spectrum was made with several characteristic features including extreme anxiety with change in routines, lack of imaginative play with repetition, and a very restricted interest in certain foods. He continued had high activity levels and inattentiveness. Due to reported absences with concentration difficulties an EEG was recommended, but it turned out to be technically difficult due to behaviour. From the age of 7 years he represented due to frequent falls where he tripped, which were initially attributed to his high activity levels and impulsivity. He was referred to paediatric neurology and investigation was recommended including an EEG which confirmed that the events were epileptic in nature and he had frequent drop attacks. An MRI scan revealed high signal in the dentate nuclei as well as heterogenous pattern of signal intensity in both thalami (figure 1). The cerebellar tonsils showed descent with prominence of the optic nerve sheaths.

On examination at the age of 8years and 2 months he was active with features consistent with autistic spectrum disorder and ADHD . He had very frequent episodes of losing tone suddenly with vacant staring episodes and occasional myoclonic jerks. He was also seen to stumble and veer several times to left side with no provoked stimulus. . The episodes of drop attacks had stopped since starting the clobazam by the local team and this was changed to sodium valproate following the EEG. The epileptic seizures had had been difficult to control until sodium valproate was introduced, but this monotherapy treatment resulted in a complete loss of appetite and severe weight loss over a six week period. Because it was difficult to obtain EEG recordings, an EEG was carried out when he was waking up from anaesthesia following the brain MRI. That recording revealed multifocal spikes over both fronto-temporal regions. His epilepsy control deteriorated over two weeks at the age of 10 years. Following the severe weight loss he was switched from valproate to lamotrigine. His appetite returned within six weeks.and he showed a good response with no further drop attacks and an improvement in frequency of absences.. He remains stable and is now resident in a unit with specialist provision for children with severe learning difficulties and autism.

*Patient* 5 was a previously healthy girl with normal development who presented with her first epileptic seizures at the age of 17 months prior to a viral disease with fever, diarrhoea, vomiting and urticarial rash. She was admitted to the hospital due to focal fittings of the left arm, hand and left side of her mouth with no loss of consciousness. She presented with *epilepsia partialis continua* (persistent focal seizures affecting left side of the body) and was treated with diazepam, lorazepam, phenytoin after which the seizures stopped for 45 min after which she started fitting again due to which she was sedated with morphine and midazolam prior to intubation and ventilatation. Left-sided focal seizures were very resistant to anticonvulsant treatment and required a thiopentone infusion and several phenobarbitone loadings until she became seizure one week later and was successfully extubated. Whilst on the ward she was found to have some involuntary movements which involved the right as well as the left side of her body and she also had periods of marked irritability due to which she had been treated benzodiazepines, chloral hydrate and trihexyphenidyl for a short period. Neurological examination prior to discharge showed generally decreased muscle tone which was more apparent in the left arm. She was fed with a nasogastric tube. A presumptive diagnosis of encephalitis was made during her stay on intensive care due to which she was initially treated also with cefuroxime and acyclovir. Apart from a positive rhinovirus in the nasopharyngeal aspirate studies on blood and CSF for common viruses were all negative. Head CT was normal. CSF protein was raised on three occasions with no white cells present, with normal glucoses and a slightly raised CSF lactate on one occasion. Neurometabolic investigations showed normal liver function tests, blood lactates and urinary organic acids. Head CT was normal and brain MRI. By the time of discharge she had no clear epileptic seizures for several weeks, but since then she developed *epilepsia partialis continua* (persistent focal seizure affecting left side of the body) despite treatment with phenobarbitone and levetiracetam in addition to involuntary movements, fluctuating right hemiparesis and visual impairment. She had a further clinical deterioration from the age of 21 months and she died at the age of 2 yrs 1 month due to intractable epilepsy, but without liver dysfunction.

***Patients (6-8) identified from a retrospective cohort of patients with POLG1 mutations and epilepsy without liver involvement at presentation***

*Patient 6* was a girl who was her parent’s first child, but who had two healthy paternal stepbrothers. She had no evidence for a movement disorder, but had a mild global developmental delay. Muscular hypotonia was noticed at the age of 15 months before the sudden onset of *status epilepticus.* The first seizure at the age of 18 months was followed by *status epilepticus* after which she remained markedly encephalopathic, having multiple seizures, and subsequently died at the age of 2 years. The parents refused a post mortem examination, but clinically there was a very strong suspicion of Alpers disease based on MRI brain changes consistent with a mitochondrial disease. The preliminary brain MRI was normal, but then basal ganglia changes and some atrophy were found. She had severe epilepsy, but no liver disease and she did not have valproate treatment. EEG recordings revealed a very disordered background with epileptiformic discharges.

*Patient 7* was well until at the age of 16 years, when she developed intermittent jerking of the left leg. Treatment was not started because clinical examination, brain MRI and EEG were all normal and there was no family history. A year later at the age of 17 yrs she was admitted with generalised tonic clonic seizures and *status epilepticus* and a tertiary referral was made. Her seizures were refractory to all antiepileptic drugs including sodium valproate. She had three admissions to intensive care for *status epilepticus* over the next nine months, and developed bilateral cortical blindness, rotatory nystagmus and lower limb weakness with loss of reflexes. CSF oligoclonal bands and other routine tests were normal on the first admission. Serial EEGs showed epileptiform activity predominantly on the right. Brain MRI on admission revealed areas of high signal in the right thalamus and left cerebellar hemisphere (Figure 2A and 2B). Repeated brain MRI two months later showed bilateral occipital lesions with improvement in the previous abnormalities (Figure 2C). A further scan 3 months later showed a new lesion in the right parietal cortex (Figure 2D). Brain MR spectroscopy showed an increased choline peak in the right frontal cortex and increased lactate peak in the posterior fossa. She then developed mildly abnormal liver function suggesting Alpers syndrome. The patient died due to intractable epilepsy 2 years after presentating with her first epileptic seizure.

*Patient 8* was a girl with normal development until 15 months when she lost some skills. At the age of 18 months she suddenly presented with *status epilepticus*. In the 2 weeks prior to the admission she had had evidence of an eye deviation and she had been admitted to hospital a week before with a “viral” infection, when she had appeared floppy and had been vomiting. After sending her back home she had her first epileptic seizure, which was treated with i.v. lorazepam after which she became apnoeic. In paediatric intensive care unit she presented with an intractable *status epilepticus* and never recovered consciousness. Her liver function tests showed mild hepatic dysfunction with a max ALT 98 IU/l. She had persistently raised plasma lactates (2-4 mmol/l) with normal acylcarnitines and plasma amino acids. Organic acids showed mildly raised 4-hydroxyphenyllactate and 4-phenylpyruvate and trace amounts of triglycine. She died within 1 month due to severe *status epilepticus* and liver failure.
